# Investigation of Synthesis Mechanism, Optimal Hot-Pressing Conditions, and Curing Behavior of Sucrose and Ammonium Dihydrogen Phosphate Adhesive

**DOI:** 10.3390/polym12010216

**Published:** 2020-01-15

**Authors:** Zhongyuan Zhao, Shunsuke Sakai, Di Wu, Zhen Chen, Nan Zhu, Chengsheng Gui, Min Zhang, Kenji Umemura, Qiang Yong

**Affiliations:** 1 College of Furnishings and Industrial Design, Nanjing Forestry University, Nanjing 210037, China; zhaozy930@126.com (Z.Z.); nfuwudi@163.com (D.W.); 2 Laboratory of Sustainable Materials, Research Institute for Sustainable Humanosphere, Kyoto University, Gokasho, Uji, Kyoto 611-0011, Japan; shunsuke_sakai@rish.kyoto-u.ac.jp (S.S.); zhang888@rish.kyoto-u.ac.jp (M.Z.); Umemura@rish.kyoto-u.ac.jp (K.U.); 3 Qingdao Institute of Bioenergy and Bioprocess Technology, Chinese Academy of Sciences, Qingdao 266101, China; chenzhen@qibebt.ac.cn; 4 Jiangsu Center of Supervision & Testing on Green Degradable Material Quality, Nanjing 210019, China; nzhuwork@126.com; 5 Zhejiang Shenghua Yunfeng Greeneo Co., Ltd., Deqing 313000, China; gcs19882006@126.com; 6 College of Chemical Engineering, Nanjing Forestry University, Nanjing 210037, China

**Keywords:** eco-friendly adhesive, sucrose, ammonium dihydrogen phosphate, plywood

## Abstract

In this study, a further investigation was carried out on the synthesis mechanism, optimal manufacturing conditions, and curing behavior of a sucrose-ammonium dihydrogen phosphate (SADP) adhesive. The results of ^13^C nuclear magnetic resonance (NMR) spectroscopy confirmed that SADP was composed of 5-hydroxymethylfurfural (5-HMF), deoxyfructosazine (DOF), amino compounds, Schiff base, monosaccharides, and oligosaccharide. The optimal hot-pressing conditions were a hot-pressing temperature of 170 °C, a hot-pressing time of 7 min, and a spread rate of 120 g/m^2^. The wet shear strength of plywood bonded at optimal manufacturing conditions met the requirements of China National Standard (GB/T 9846-2015). Thermal analysis and insoluble mass proportion measurements showed that the main curing behavior of the SADP adhesive occurred at curing temperatures higher than 145 °C, and more than 50% insoluble mass was formed when the heating time was longer than 5 min. Fourier-transform infrared spectroscopy (FT-IR) indicated that cross-linking of the cured adhesive was promoted by prolonging the heating time. In addition, pyrolysis gas chromatography and mass spectrometry (Py-GC/MS) confirmed that the cured SADP adhesive was composed of furan and nitrogen-containing compounds.

## 1. Introduction

Due to decreasing fossil fuel resources and rapidly increasing environmental awareness, the utilization of renewable resources has become a focus of materials science research [1,2,3,4]. As a consequence, many novel bio-based materials have been developed and are expected to be utilized in many fields [5,6,7,8,9,10]. Wood-based materials (such as particleboard [11], plywood [12], oriented strand board [13], and fiberboard [14]) are common in everyday living environments, and are typically fabricated using formaldehyde-based resins to obtain excellent bonding performance [15,16,17]. However, formaldehyde-based resins have been confirmed to emit free formaldehyde during utilization, which is known to negatively affect human health [18,19]. Therefore, it is necessary to develop an adhesive based on renewable and nontoxic resources. 

Sucrose is one of the most abundant renewable resources and is available at very high levels of purity at a low cost. As a result, this natural product has been extensively used as a raw material and is widely used in the beverage and food industries [20,21]. We have previously demonstrated the use of sucrose to prepare eco-friendly adhesives for wood-based materials, such as sucrose-citric acid [22], sucrose-tannin [23], and sucrose-acid-tannin adhesives [24]. In our recent research, a novel sucrose-based adhesive was explored in which sucrose and ammonium dihydrogen phosphate (ADP) were dissolved in distilled water at room temperature and successfully applied to a particleboard product [25]. The results suggested that this type of adhesive system might be suitable for use in a variety of wood composite products.

A highly concentrated solution of sucrose with relatively low viscosity was obtained by heating sucrose in a small amount of distilled water [20,21], which made it possible to synthesize a sucrose-based adhesive with higher solid content and a viscosity that was appropriate for wood product manufacturing. Therefore, to explore the application of sucrose-ammonium dihydrogen phosphate (SADP) adhesives, we developed a material that can be synthesized with minimal water with a high solids content and suitable viscosities for introduction into existing plywood manufacturing operations. The optimal synthetic conditions and curing mechanism have also been clarified [26,27]. To further understand the mechanism and curing behavior, which was helpful to optimize this novel adhesive, on this study, a further research on the synthetic mechanism, optimal hot-pressing conditions, and curing behavior will be performed.

## 2. Materials and Methods 

### 2.1. Materials 

Sucrose (analytical-grade reagent) and ammonium dihydrogen phosphate (analytical-grade reagent) were purchased from Sinopharm Chemical Reagent Co., Ltd. (Shanghai, China). Each reagent was vacuum-dried at 60 °C until constant mass prior to usage in experiments. Poplar veneers were obtained from Zuogezhuang, Hebei Province of China.

### 2.2. Synthetic Procedures of SADP Adhesive

The synthesis method of SADP adhesive was followed the previous research [27], and the detail information was described as follows: sucrose and ammonium dihydrogen phosphate (ADP) were mixed at 90/10 mass proportions, and were poured into a three mouthed flask with distilled water to synthesize adhesive with 80 wt.% solid content. The mixture was then heated in an oil bath at 90 °C for 3 h, while it was sheared at 180 rpm/min to facilitate synthesis of the sucrose-ammonium dihydrogen phosphate (SADP) adhesive. The pH of the adhesive was measured at 30 °C using a Leici pH meter PHBJ-206 (Leici, Shanghai, China), and the viscosity of the adhesive was measured using a HAAKE rotational rheometer MA S60 (HAAKE Co., Karlsruhe, Germany). The results of pH and viscosity of SADP adhesive were 3.7 and 826.7 mPa·s, respectively. Synthesized SADP adhesive was sealed and stored at room temperature at least 3 days for further research. 

### 2.3. ^13^C Nuclear Magnetic Resonance (NMR) Analysis

^13^C NMR spectra were acquired on a Bruker AVANCE 600 MHz spectrometer equipped with a 5 mm BBO probe using an inverse gated proton decoupling sequence. An amount of 100 mg of freeze-dried SADP adhesive was dissolved in 0.5 mL DMSO-*d*_6_. Then, the solution was transferred to the Shigemi microtube and characterized at 25 °C. The acquisition parameters were 90° pulse width, a relaxation delay of 1.7 s, and an acquisition time of 1.2 s. A total of 10,000 scans were collected.

### 2.4. Effects of Hot Pressing Conditions on the Bond Performance of SADP Adhesive

#### 2.4.1. Manufacture of Plywood

The synthesized SADP adhesives were utilized to manufacture a three-layer plywood (300 mm × 300 mm), the bond performance of which was evaluated. The moisture content and thickness of the veneers were 9.8–11% and 1.5 mm, respectively. SADP adhesive was applied to the double surfaces of core veneer. The coated veneer was stacked between two uncoated veneers so that the grain direction of both adjacent veneers was perpendicular to each other. All assembled three-layered plywood samples with SADP adhesive were hot-pressed at three groups manufacture conditions to investigate the effects of hot pressing temperature, hot pressing time, and spread rate on the bond performance. The detailed information of these conditions is shown in Table 1. 

#### 2.4.2. Shear Strength Measurement

The prepared plywoods were cut into standard tensile shear test specimens according to China National Standards (GB/T 9846.7-2004). Twelve plywood specimens (10 cm × 2.5 cm) were cut from each manufactured plywood and six specimens were submerged into water at 63 ± 2 °C for 3 h, and then, the tensile shear strength of the plywood were measured at dry and wet conditions under a loading rate of 1.0 mm/min. Each plywood test was carried out in twelve replications, and the average values, standard deviations, and average wood failure were calculated. Statistical significance was considered for *p* values < 0.05.

### 2.5. Curing Behaviour of SADP Adhesive

#### 2.5.1. Thermal Analysis

One part of synthesized SADP adhesive were poured into glass vials and then frozen by a refrigerator. Following complete freezing, the samples were dried using a laboratory lyophilizer to obtain an uncured samples, which would be analyzed by thermogravimetric analysis (TGA) and differential scanning calorimetry (DSC) using a Discovery TGA 55 (TA Instruments, Tokyo, Japan) and Discovery DSC 25 (TA Instruments, Tokyo, Japan), respectively. The samples were scanned from room temperature to 400 °C at a rate of 10 °C/min under nitrogen purging with the flow rate at 70 mL/min and 50 mL/min, respectively. In addition, DSC results exhibited without mass of the sample at a given temperature, but initial mass of the sample.

#### 2.5.2. Insoluble Mass Proportion 

A part of uncured SADP adhesive was divided into two groups, and each group of the uncured SADP adhesive was further separated into four parts. In Group 1, each part of uncured SADP adhesives were heated at 130, 150, 170, and 190 °C for 7 min, respectively; in Group 2, the samples were heated at 190 °C for 3, 5, 7, and 9 min to prepare cured adhesive samples. Approximate 2 g of each cured sample were then boiled in distilled water for 4 h to obtain insoluble mass, an act that was carried out in triplicate. The obtained insoluble mass were vacuum-dried at 60 °C until constant mass (15 h) and finally weighed. The insoluble mass proportion was calculated by the following equation:

(1) $$\mathrm{Insoluble}\;\mathrm{mass}\;\mathrm{proportion}\;\left(\%\right)=\frac{Weight\;of\;dried\;insoluble\;mass\;\left(g\right)}{Weight\;of\;heated\;sample\left(g\right)}\;\times 100\%.$$

#### 2.5.3. Fourier Transform Infrared Spectra (FT-IR)

Fourier transform infrared spectra were acquired to investigate the effects of heating time on the chemical changes of insoluble mass of SADP adhesive (cured at 170 °C for 5, 7, and 9 min, and after the boiling treatment). Infrared spectra were obtained using a Fourier transform infrared spectrophotometer (FT/IR-4200, JASCO Corporation, Tokyo, Japan) by the KBr disk method, and recorded with an average of 32 scans at a resolution of 4 cm^−1^.

#### 2.5.4. Pyrolysis Gas Chromatography and Mass Spectrometry (Py-GC/MS)

Pyrolysis gas chromatography and mass spectrometry (Py-GC/MS) was employed to clarify the chemical composition of cured SADP adhesive. The insoluble mass of cured SADP adhesive (cured at 170 °C for 7 min, and after boiling treatment) was pyrolyzed at 500 °C with 60 s by Multi-Shot pyrolyzer (EGA/PY-3030d, Frontier Laboratories Ltd., Koriyama, Japan), and the pyroprobe interface temperature was set at 400 °C. The volatile compounds during the heating process were measured by a Py-GC/MS system (GCMS-QP2010, Shimadzu Co., Ltd., Kyoto, Japan), and the column was an Ultra ALLOY-5 capillary column (30 m × 0.25 mm i.d., 0.25 μm film thickness, Frontier Laboratories Ltd., Fukushima, Japan). The initial temperature of the column was set at 37 °C for 2 min, and then, the temperature was increased to 100 °C with a rate of 30 °C/min, immediately the temperature was further increased to 300 °C at a rate of 15 °C/min and kept at 300 °C for 1 min. The mass spectrometer was operated in EI mode at 70 eV, and analyzed the range was 50–600 m/z with a scan speed of 1250 amu/s. The pyrolysis products were identified using the NIST 08 mass spectral library, and the identified volatile components with highest and greater than 80 similarity index (SI) were recorded.

## 3. Results and Discussion

### 3.1. Synthesis Mechanism of SADP Adhesive

**Figure 1:**
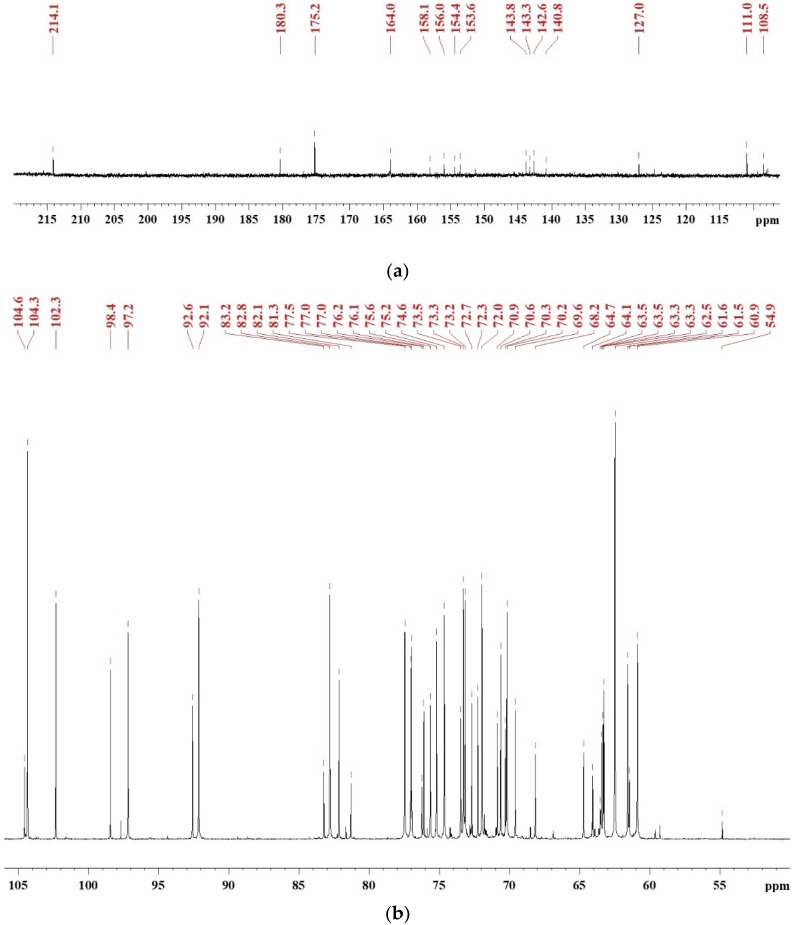
^13^C NMR spectrum of freeze-dried SADP adhesive in DMSO-*d*_6_: (**a**) chemical shifts in the region of 220–106 ppm and (**b**) the chemical shifts in the region of 106–50 ppm.

To investigate the synthesis mechanism of SADP adhesive, the ^13^C NMR spectra (Figure 1) were recorded to serve as an information source, and to supplement a previous discussion [26,27]. To clearly identify chemical shifts, the NMR spectra were divided to two regions: 220–106 ppm (Figure 1a) and 106–50 ppm (Figure 1b). The peak located at 214.4 ppm was attributed to a C=O bond of ketones [28], which formed during the dehydration of sucrose [29]. The chemical shifts located at 180.3, 164.0, 127.0, 111.0, 153.6, and 54.9 ppm were assigned to the C6, C5, C4, C3, C2, and C1 of 5-HMF [30,31,32], respectively, and this result has also been clarified by HPLC in previous research [26,27]. The peaks from 154.4 to 158.1 ppm and 140.87 to 143.8 ppm were attributed to C2–C5 of 2,6-deoxyfructosazine (2,6-DOF) and 2,5-deoxyfructosazine(2,5-DOF), respectively [33,34]. The chemical shift at 175 ppm was due to the amide bond, and the signal at 164 ppm was also attributed to the C=N of Schiff base [35]. A dense region at 60–104 ppm containing many peaks was due to monosaccharides (such as glucose, fructose, and their isomers) and oligosaccharide [36,37,38,39]. Combining pH measurements and NMR analysis, it could be clarified that the synthesized SADP adhesive was a mixture composed of monosaccharides, ketones, Schiff base, oligosaccharide, deoxyfructosazine, and acid. 

**Figure 2:**
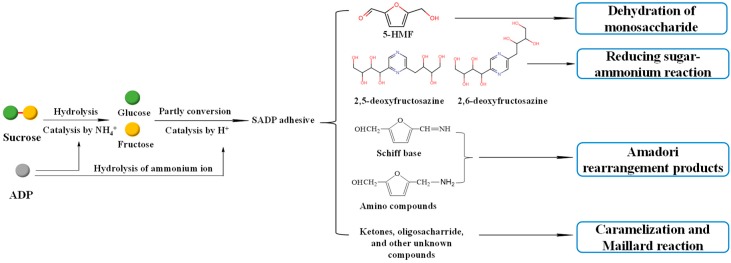
Possible synthesis mechanism of SADP adhesive.

The proposed synthesis mechanism is shown in Figure 2, which shows that the synthesis of the SADP adhesive is a complex reaction. During heat treatment, ammonium catalyzed the hydrolysis of sucrose to glucose and fructose, and the ammonium ion from ADP also was hydrolyzed to ammonium hydroxide [40]. Some of the liberated hexoses were converted to 5-HMF, and some of the 5-HMF reacted with ammonium hydroxide and formed amino compounds and Schiff base via an Amadori rearrangement. In addition, 2,5-DOF and 2,6-DOF were formed from the reaction of a reducing sugar and ammonium [41]. The ketones, oligosaccharides, and other unknown compounds were also present in the synthesized SADP adhesive, and their formation occurred via caramelization and Maillard reactions [42,43].

### 3.2. Effects of Hot Pressing Conditions on the Bond Performance

**Figure 3:**
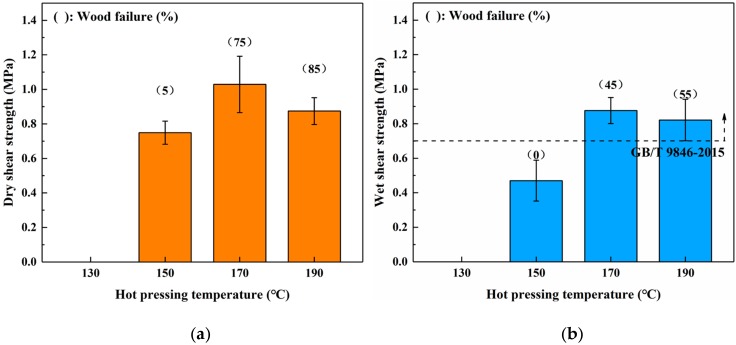
Effects of hot pressing temperature on the bond performance of plywood: (**a**) dry shear strength and (**b**) wet shear strength.

To investigate the effects of different hot-pressing conditions on the bond performance of the SADP adhesive, plywood was fabricated at various hot-pressing temperatures, hot-pressing times, and spread rates. Figure 3 shows the dry and wet strength of plywood bonded from 130 to 190 °C. The board hot-pressed at 130 °C exhibited no adhesion strength, indicating the SADP curing reaction was insufficient at this temperature. As the hot-pressing temperature increased from 150 to 170 °C, both the (dry and wet) shear strength and wood failure increased. The maximum dry and wet shear strengths were obtained from the plywood bonded at 170 °C (1.03 and 0.88 MPa). However, when the hot-pressing temperature increased to 190 °C, a decrease in the shear strength, as well as an increase in wood failure, were observed. This phenomenon was attributed to a decrease in the veneer strength, which was caused by the acidity of SADP adhesive, and the acid compounds deteriorated the wood elements at high pressing temperatures [44]. The wet shear strength of boards bonded at 170 and 190 °C met the requirements of China National Standard (GB/T 9846-2015). Since the maximum shear strength was obtained from the plywood bonded at a lower hot-pressing temperature, 170 °C was selected as the optimal hot-pressing temperature.

**Figure 4:**
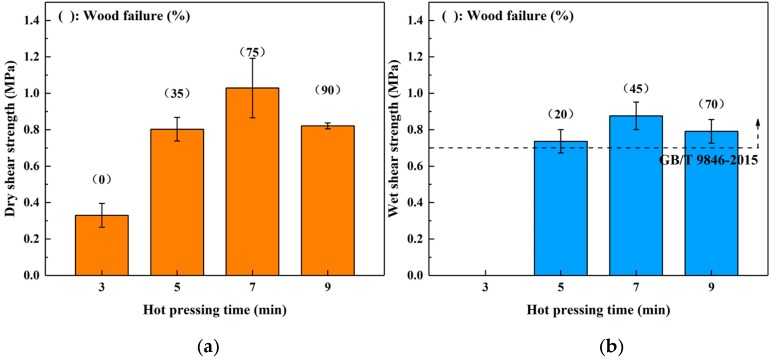
Effects of hot pressing time on the bond performance of plywood: (**a**) dry shear strength and (**b**) wet shear strength.

The profiles shown in Figure 4 show the dry (a) and wet (b) shear strength of the boards bonded for different hot-pressing time. In the dry shear strength, the bond performance was promoted by increasing the hot-pressing time from 3 to 7 min (the maximum value was 1.03 MPa). However, a decreasing trend was observed when the hot-pressing time was increased to 9 min, and the wood failure also increased. This phenomenon was similar to the trend observed when varying the hot-pressing temperature above, which was also attributed to the acidity of the SADP adhesive. The boards bonded for 3 min did not maintain their original shape during immersion treatment, indicating the curing reaction was incomplete using this heating time. When the heating time was longer than 5 min, the wet shear strength of all the samples bonded by the SADP adhesive exceeded the requirement of China National Standard (GB/T 9846-2015). Since the minimum value of the error bar of the boards bonded for 5 min was lower than the requirement of the standard (0.7 MPa), the optimal hot-pressing time was determined to be 7 min.

**Figure 5:**
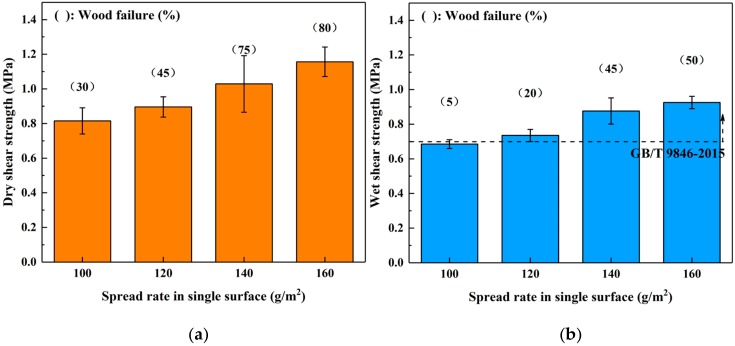
Effects of spread rate in single surface on the bond performance of plywood: (**a**) dry shear strength and (**b**) wet shear strength.

The influence of spread rate on the bond performance of SADP adhesive was investigated by manufacturing plywood samples at 170 °C for 7 min with a 100–160 g/m^2^ spread rate on a single surface. The results shown in Figure 5 show that both the (dry and wet) shear strength and wood failure were improved as the spread rate increased, indicating that the bond performance of SADP was positively correlated with the spread rate. In addition, the wet shear strength of the boards bonded using spread rates equal to or higher than 120 g/m^2^ satisfied the requirements of China National Standard (GB/T 9846-2015). Since the wet shear strength of the plywood bonded with a 120 g/m^2^ spread rate was 0.74 MPa, which exceeded the standard, the optimal spread rate was determined to be 120 g/m^2^.

Based on the investigation of bond performance of the SADP adhesive above, the optimal manufacturing conditions were determined to be: a hot-pressing temperature of 170 °C, hot-pressing time of 7 min, and a spread rate of 120 g/m^2^. However, compared with industrial conditions, these fabrication conditions are harsher than those used for synthetic resins. Therefore, the curing behavior and chemical analysis should be confirmed, which will be helpful to explore methods to reduce the manufacturing costs of such materials.

### 3.3. Curing Behavior of SADP Adhesive

#### 3.3.1. Thermal Analysis

**Figure 6:**
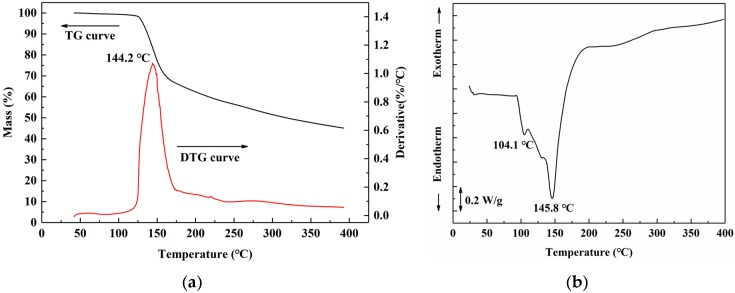
(**a**) Thermo gravimetric (TG) and derivative thermo gravimetry (DTG) curves, and (**b**) differential scanning calorimetry (DSC) curve of SADP adhesive.

Thermal analysis of freeze-dried uncured SADP adhesive was performed to clarify the curing behavior during heating treatment. The thermo gravimetric (TG) results (Figure 6a) showed that SADP exhibited a preliminary mass loss near 125 °C. In the derivative thermo gravimetry (DTG) curve, a one-step thermal degradation was observed, and rapid weight loss occurred near 144.2 °C. Previous research indicates that sucrose undergoes a two-step degradation process. The first step occurs near 223 °C, which is attributed to the caramelization of sucrose [45]; the second step occurs near 267 °C, which is due to the formation of a black, aerated, and char-like solid [46]. A significant reduction in the thermal degradation temperature was observed in the SADP adhesive, due to catalysis of ADP during sucrose degradation or curing reaction between sucrose and ADP. The DSC profile (Figure 6b) of SADP displayed a shoulder at 104.4 °C and an endothermic peak at 145.8 °C. The TG analysis indicates that thermal degradation did not occur near 104 °C; therefore, the shoulder at 104.4 °C was attributed to the melting of freeze-dried uncured SADP adhesive. In addition, the endothermic peak at 145.8 °C was similar to the rapid thermal degradation temperature (144.2 °C), indicating that the main curing behavior of SADP occurred near 145 °C. 

#### 3.3.2. Insoluble Mass Proportion

**Figure 7:**
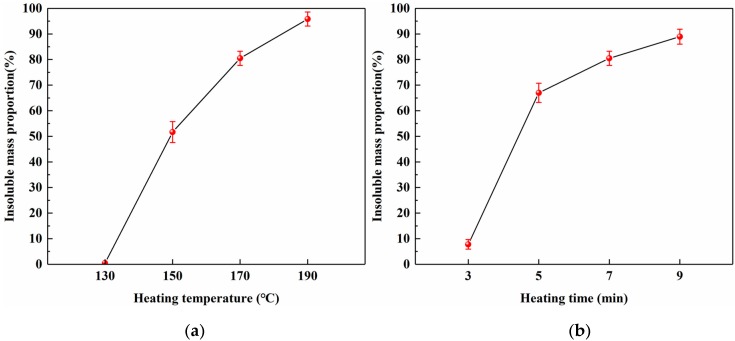
Effects of (**a**) heating temperature and (**b**) heating time on the insoluble mass proportion.

To investigate the relationship between the heating conditions and curing behavior of SADP, the insoluble mass proportion was measured. Figure 7a shows a nearly linear increase in the insoluble mass proportion as the heating temperature increased (heating time: 7 min), indicating a positive correlation between the temperature and curing of SADP. The highest value (95.8%) was obtained at 190 °C, implying that the curing at this temperature was extremely sufficient. When the heating temperatures were 150 and 170 °C, the insoluble mass proportions were 51.6% and 80.5%, respectively, demonstrating that the majority of SADP had been cured at these temperatures. The thermal analysis results above indicate that the main curing behavior of SADP adhesive occurred near 145 °C; therefore, the mass loss proportion at 130 °C was nearly zero (0.47%). Figure 7b shows the effects of heating time on the curing process of SADP, which exhibited a positive correlation between the heating time and insoluble mass proportion (heating temperature: 170 °C). An obvious rapid increase was observed when the heating time increased from 3 (7.8%) to 5 min (67.0%), implying that the curing of SADP required a certain heating time. At heating times longer than 5 min, more than 60% of the SADP adhesive was cured. 

#### 3.3.3. Chemical Change

**Figure 8:**
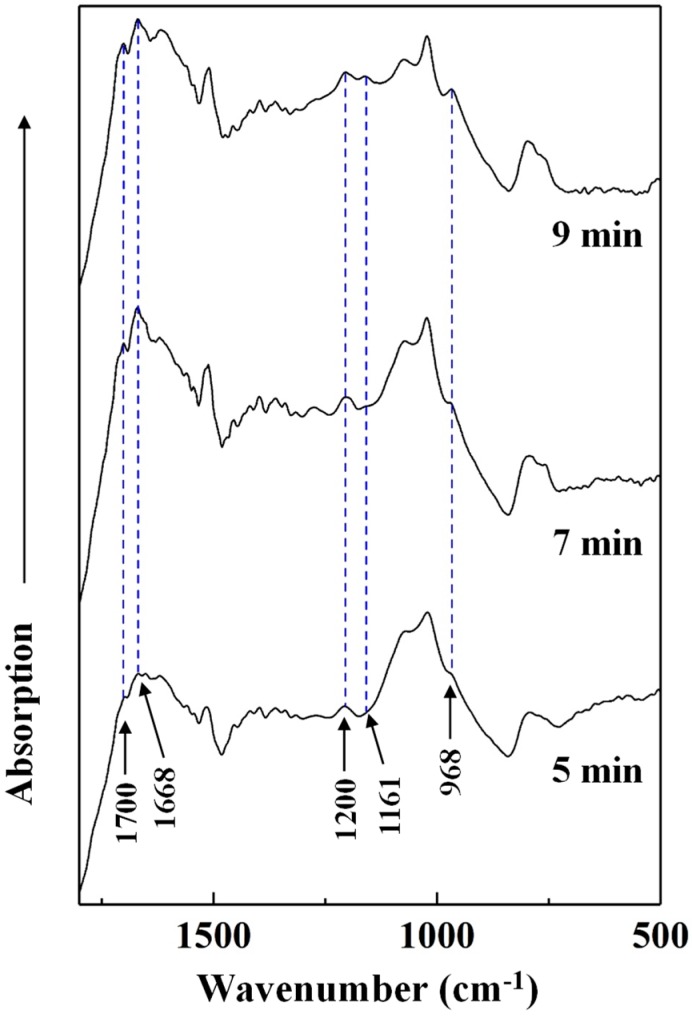
FT-IR spectra of insoluble mass derived from SADP adhesive heated for different time.

To determine the effects of heating conditions on the chemical changes of the cured adhesive, FT-IR spectra of the insoluble mass were recorded (Figure 8). Since the effects of heating temperature on the chemical structure of cured SADP has been studied in our previous research [26], we only analyzed the influence of heating time in this study. Figure 8 shows the absorption bands of the insoluble mass heated at 170 °C for 5, 7, and 9 min. As the heating time was prolonged, the intensity of five peaks located at 1700, 1668, 1200, 1161, and 968 cm^−1^ increased. The peak located at 1700 cm^−1^ was attributed to a carbonyl group [47]. The absorption bands at 1668 and 1161 cm^−1^ were attributed to a C=N group of an imine and C–N bonds [48,49]. In addition, the peaks at 1200 and 968 cm^−1^ were attributed to C–O–C stretching and C–O symmetric stretching vibrations [50,51]. The increase in these groups indicated that prolonging the heating time helped ensure more complete cross-linking (imine and dimethylene ether bridges) in the cured SADP adhesive.

### 3.4. Chemical Composition of Cured SADP Adhesive

**Figure 9:**
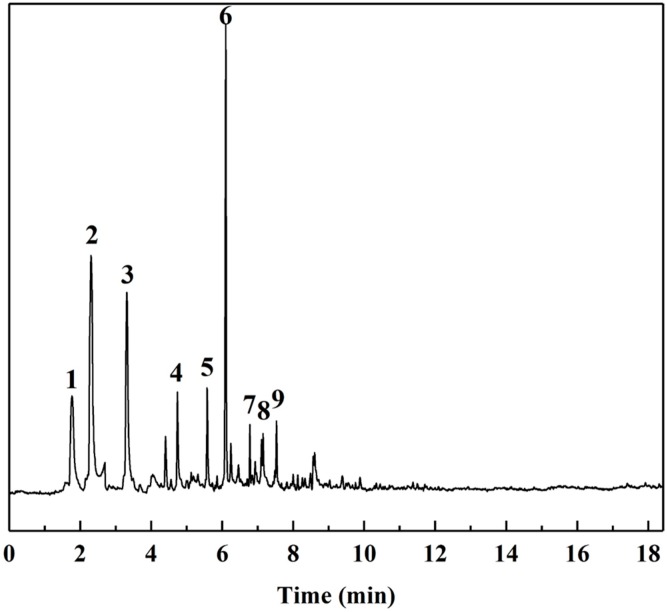
GC/MS chromatogram of evolved gas taken from the insoluble mass of cured adhesive heated at 500 °C for 60 s.

**Figure 10:**
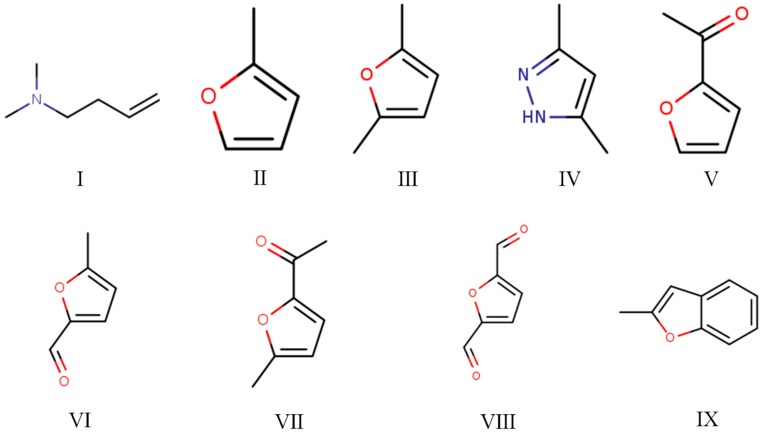
Chemical structure of the identified compounds in the evolved gas derived from the insoluble mass of cured SADP adhesive heated at 500 °C for 60 s.

The Py-GC/MS chromatograms (Figure 9) were obtained to determine the chemical composition of cured SADP adhesive by pyrolyzing the insoluble mass derived from SADP adhesive (heated at 170 °C for 7 min) at 500 °C for 60 s. The pyrolysis data and chemical structure of the identified compounds listed in Table 2 and Figure 10 show that most of the pyrolyzed products contained a furan ring. Substances III, VI, VII, and VIII indicated that 5-HMF was the main monomer in the cured adhesive, and other furan compounds containing one substituent (substances II, V, and IX) were derived from the pyrolysis products of sucrose (such as furfural) and also participated in the curing reaction. Pyrolysis products V, VI, VII, and VIII contain carbonyl groups at the terminal positions their molecules, indicating that C–O–C was the main cross-linkage in the cured adhesive. In addition, the peaks located at 1.76 and 4.74 min (substances I and IV) were taken as evidence that nitrogen-containing compounds participated in the curing reaction. Combined with the analysis of synthesis mechanism above (Figure 1 and Figure 2), these compounds were possibly derived from the formation of Schiff base and heterocyclic compounds. The results of Py-GC/MS confirmed that the cured SADP adhesive contains furan and nitrogen-containing compounds.

## 4. Conclusions

Sucrose and ammonium dihydrogen phosphate were utilized to synthesize an eco-friendly adhesive (SADP) for plywood. The ^13^C NMR results confirmed that the synthesized SADP adhesive was composed of 5-hydroxymethylfurfural (5-HMF), deoxyfructosazine (DOF), amino compounds, Schiff base, monosaccharides, and oligosaccharide. The formation of these chemical compounds was related to the hydrolysis of sucrose and ammonium ion, the dehydration of monosaccharides, the Amadori rearrangement, caramelization, and the Maillard reaction. The investigation of the bond performance of plywood bonded with SADP at different manufacturing conditions showed that the optimal hot-pressing temperature, hot-pressing time, and spread rate were 170 °C, 7 min, and 120 g/m^2^, respectively. When the plywood was bonded at these optimal hot-pressing conditions, its wet shear strength met the value specified by the China National Standard GB/T 9846-2015. The curing behavior of the SADP adhesive was studied by thermal analysis, insoluble mass proportion, and FT-IR analysis. Compared with using only sucrose, the TGA and DSC curves of SADP showed a lower thermal degradation and an endothermic reaction (near 145 °C). In addition, as the heating temperature increased higher than 150 °C and at a heating time longer than 5 min, the insoluble mass proportion exceeded 50%. FT-IR spectra indicated that prolonging the heating time helped establish significant cross-links in the cured SADP adhesive. To further clarify the chemical composition of cured adhesives, Py-GC/MS was carried out, and the results confirmed that cured SADP contained furan and nitrogen-containing compounds. Based on the synthesis and curing mechanisms, methods to reduce the hot-pressing temperature and time will be studied in further research.

## Figures and Tables

**Table 1:** Manufacture conditions of the plywood bonded by SADP adhesive.

Groups	Hot Pressing Temperature (°C)	Hot Pressing Time (min)	Spread Rate (g/m^2^)
**Group 1**	130	7	140
	150		
	170		
	190		
**Group 2**	170	3	140
		5	
		7	
		9	
**Group 3**	170	7	100
			120
			140
			160

**Table 2:** Identified chemical compounds in evolved gas derived from insoluble mass of cured SADP adhesive heated at 500 °C for 60 s.

Peak Number	RT (min)	SI	Compound	CAS	MW	Formula	Chemical Structure Number
1	1.76	89	*N,N*-dimethyl(3-butenyl)amine	55831-89-5	99	C_6_H_13_N	I
2	2.30	96	2-Methylfuran	534-22-5	82	C_5_H_6_O	II
3	3.31	97	2,5-Dimethylfuran	625-86-5	96	C_6_H_8_O	III
4	4.74	83	3,5-Dimethylpyrazole (DMP)	67-51-6	96	C_5_H_8_N_2_	IV
5	5.58	96	2-Acetylfuran	1192-62-7	110	C_6_H_6_O_2_	V
6	6.10	97	5-Methylfurfural	620-02-0	110	C_6_H_6_O_2_	VI
7	6.78	96	2-Acetyl-5-methylfuran	1193-79-9	124	C_7_H_8_O_2_	VII
8	7.15	90	2,5-Furandicarboxaldehyde	823-82-5	124	C_6_H_4_O_3_	VIII
9	7.53	87	2-Methylbenzofuran	4265-25-2	132	C_9_H_8_O	IX
